# Laparoscopic Partial Cystectomy for Inflammatory Myofibroblastic Tumor of the Urinary Bladder

**DOI:** 10.1002/iju5.70100

**Published:** 2025-10-16

**Authors:** Hajime Yamasaki, Shigeaki Nakazawa, Kentaro Takezawa, Taigo Kato, Koji Hatano, Yoichi Kakuta, Atsunari Kawashima, Shinichiro Fukuhara, Norio Nonomura

**Affiliations:** ^1^ Department of Urology, Graduate School of Medicine The University of Osaka Suita Osaka Japan

**Keywords:** bladder, inflammatory myofibroblastic tumor (IMT), laparoscopic partial cystectomy

## Abstract

**Introduction:**

Inflammatory myofibroblastic tumor (IMT) of the bladder is a rare benign tumor characterized by atypical spindle cell proliferation and inflammatory cell infiltration, typically involving lymphocytes and plasma cells.

**Case Presentation:**

A 38‐year‐old woman presented with micturition pain and urinary frequency. Cystoscopy revealed an elevated tumor with edematous mucosa on the anterior bladder wall. Transurethral resection confirmed IMT of the bladder but was incomplete, prompting laparoscopic partial cystectomy with cystoscopy guidance. At 2‐year follow‐up, the patient remained asymptomatic with no recurrence.

**Conclusion:**

Complete resection is the standard treatment for bladder IMT. When the tumor extends beyond the bladder wall, laparoscopic partial cystectomy with cystoscopy guidance offers a safe and effective surgical approach for achieving complete resection with adequate margins.

**Summary**

- Laparoscopic partial cystectomy was performed for the inflammatory myofibroblastic tumor of the bladder.

**Abbreviations**
ALK: anaplastic lymphoma kinase
IMT: inflammatory myofibroblastic tumor of the bladder
MRI: magnetic resonance imaging
NSAIDs: nonsteroidal anti‐inflammatory drugs
SMA: smooth muscle actin
TUR: transurethral resection

## 1 Introduction

Inflammatory myofibroblastic tumor (IMT) is a rare mesenchymal neoplasm of intermediate biological potential, first described in the bladder in 1980. Since then, just over 100 cases have been reported [1]. We present a case of bladder IMT treated with laparoscopic partial cystectomy and review relevant Japanese cases.

## 2 Case Presentation

**FIGURE 1:**
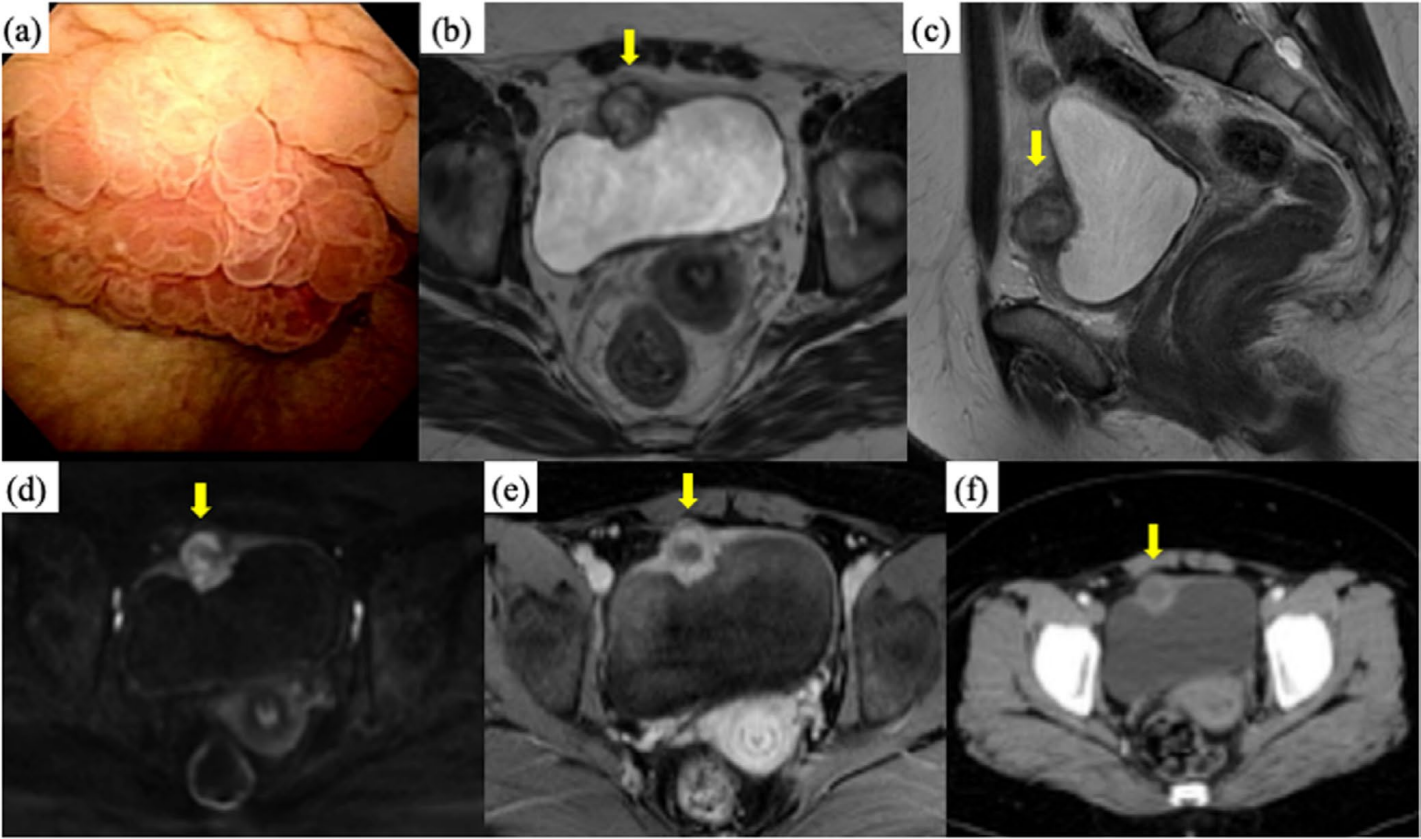
Cystoscopy showed an edematous tumor at the anterior wall of the bladder (a). Bladder mass with iso‐signal on T2‐weighted magnetic resonance imaging (b, c). Diffusion‐weighted imaging (DWI) and contrast‐enhanced MRI showed ring‐like or peripheral enhancement (d, e). Contrast‐enhanced CT exhibited similar characteristics (f).

A 38‐year‐old woman presented with micturition pain and increased urinary frequency. NSAIDs prescribed by a previous physician were ineffective. Urinalysis showed bacteriuria without pyuria or hematuria, and urine cytology was negative. Cystoscopy revealed a 20 mm elevated mass with mucosal edema on the anterior bladder wall (Figure 1a). MRI revealed a 23 mm mass invading the muscle layer (Figure 1b,c). Diffusion‐weighted imaging (DWI) and contrast‐enhanced MRI showed ring‐like or peripheral enhancement (Figure 1d,e), and contrast‐enhanced CT exhibited similar characteristics (Figure 1f). No lymphadenopathy or evidence of metastatic spread was observed.

**FIGURE 2:**
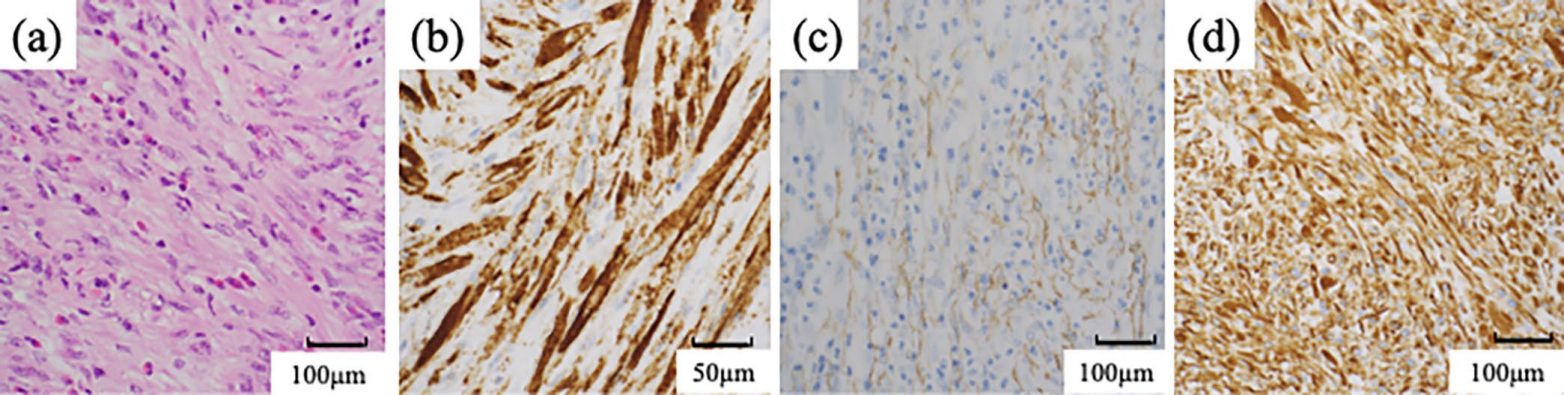
Pathological findings. (a) HE staining. Proliferation of spindle‐shaped myofibroblasts and lymphocytes, plasma cells, and other inflammatory cell infiltration. (b) ALK‐positive. (c) αSMA‐positive. (d) Vimentin‐positive.

Differential diagnoses included urachal carcinoma, urothelial carcinoma, paraganglioma, sarcomas, and benign inflammatory tumors. Paraganglioma was excluded based on endocrine tests. TUR was performed for histologic confirmation, as total cystectomy may be necessary for urachal carcinoma. Histopathology confirmed IMT based on spindle cell proliferation and inflammatory cell infiltration, with positive immunostaining for anaplastic lymphoma kinase (ALK), αSMA, and vimentin (Figure 2).

**FIGURE 3:**
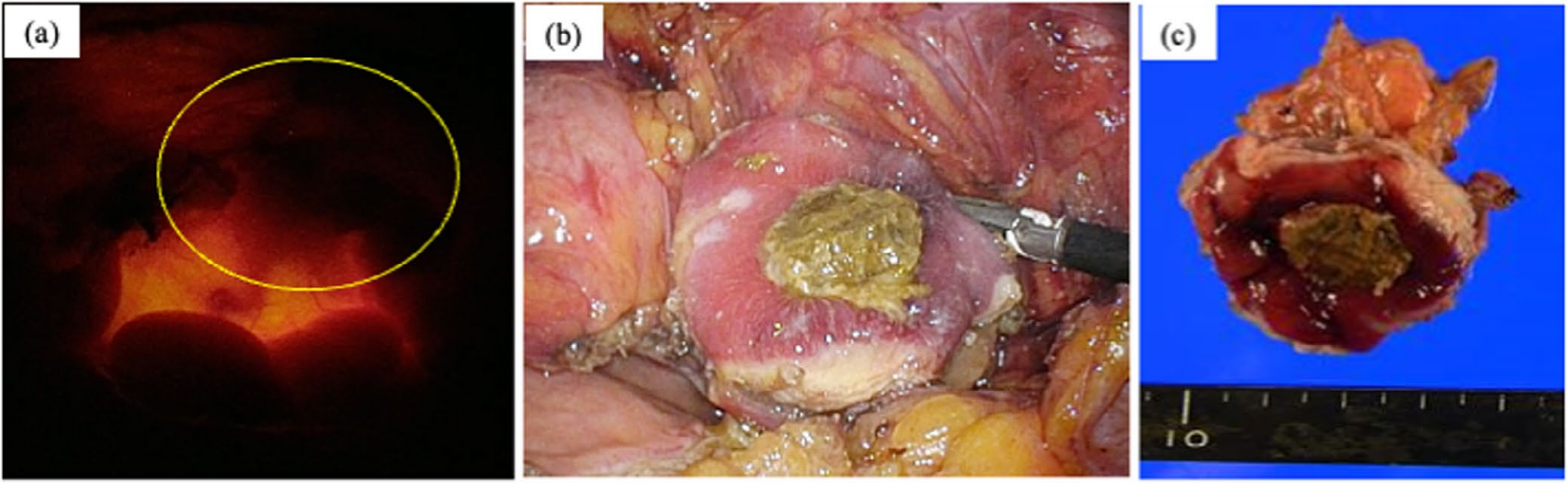
(a) Using the light of the cystoscope, the resection line was determined. (b) The tumor was resected with sufficient margins. (c) Resected specimen.

Due to extravesical extension, complete TUR was not feasible. Thus, laparoscopic partial cystectomy was performed using a three‐port technique (one 12‐mm camera port at the umbilicus and two 5‐mm working ports). Concurrent cystoscopy was performed for simultaneous intravesical visualization of the tumor; its light source enabled precise identification of the resection site. The bladder was incised laparoscopically and resected with adequate margins (Figure 3a–c). The bladder wall was closed with continuous 3‐0 barbed sutures. The patient experienced symptom resolution 2 months postoperatively. The follow‐up was carried out by an MRI imaging at 1, 4, 11, and 16 months after surgery, and the patient remained recurrence‐free for 2 years.

## 3 Discussion

IMT is a rare tumor characterized by the proliferation of spindle‐shaped myofibroblasts and infiltration of inflammatory cells such as lymphocytes, plasma cells, and eosinophils, comprising < 1% of all bladder tumor [2]. The pathogenesis remains controversial and poorly understood, but several key mechanisms have been proposed. Chronic inflammation caused by trauma, infection, or prior surgery may be involved, given the histological resemblance to granulation tissue [3]. Despite initially being regarded as a reactive lesion, increasing evidence supports a neoplastic origin. Chromosomal rearrangements involving the ALK gene on chromosome 2p23 are observed in a substantial proportion of IMTs and result in ALK protein overexpression, indicating clonal proliferation [4].

IMT must be distinguished from malignant soft tissue tumors, especially rhabdomyosarcoma and leiomyosarcoma. ALK expression is found in 33%–89% of IMTs [5, 6], although it is not entirely specific. In contrast, rhabdomyosarcomas express MyoD1 and myogenin, which help in differentiation [7].

Historically, IMT has been described by several terms, including inflammatory pseudotumor, plasma cell granuloma, pseudosarcomatous fibromyxoid tumor, and nodular fasciitis. The World Health Organization classifies IMT as a tumor of intermediate biological potential due to its low rate of recurrence and lack of distant metastasis. Bladder IMT affects patients of all ages, including pediatric cases, which account for approximately 25%. There is no strong gender predilection, although some studies suggest a slight female dominance. Symptoms include hematuria, dysuria, frequent urination, and lower abdominal discomfort [8].

Preoperative diagnosis is often difficult due to nonspecific imaging features. On MRI or CT, IMT may show ring‐like or peripheral enhancement [9] and restricted diffusion on DWI with low ADC values, suggesting high cellularity. Imaging alone cannot reliably distinguish IMT from malignant tumors such as urothelial carcinoma or sarcomas. Thus, TUR remains essential for histological confirmation.

Although IMT is benign, incomplete resection is a known risk factor for local recurrence. If TUR is insufficient for complete removal, partial cystectomy may be necessary [10]. Diode laser resection has also been reported as an effective method [11]. Furthermore, ALK‐positive IMTs may benefit from ALK inhibitor therapy, such as crizotinib, particularly in unresectable or recurrent cases [12, 13].

A review of Japanese IMT cases (*n* = 129) is shown in Table 1 [13, 14]. The median age was 43 years, and the median tumor size was 30 mm. ALK positivity was reported in 81%. Prednisolone was administered in 12 cases, with tumor shrinkage observed in 8. While no recurrences or metastases were reported in Japan, international reports noted five recurrences among 120 cases, all local [10]. These findings emphasize the importance of complete resection. We recommend postoperative follow‐up similar to that for bladder cancer, including cystoscopy and imaging studies.

**TABLE 1:** Reported cases of bladder IMT in Japan.

	*N* = 129
Age, year	43 (0–89)
Sex F/M	70/59
Tumor size, mm	30 (10–100)
Number of tumor	1
Immunohistochemical staining	
ALK‐positive	35/43 (81%)
SMA‐positive	31/35 (89%)
Steroid therapy	
Effective	8/12 (67%)
Ineffective	4/12 (23%)
Surgical treatment	
TURBT only	50
TURBT + partial cystectomy	40
TURBT + total cystectomy	12

## 4 Conclusion

We report a rare case of bladder IMT successfully treated by laparoscopic partial cystectomy. Accurate localization using cystoscopy allowed complete resection. When the tumor extends beyond the bladder wall, laparoscopic partial cystectomy is an effective and minimally invasive option. Complete surgical excision remains the cornerstone of treatment.

## Consent

Written informed consent was obtained from the patient for the publication of this case report and the accompanying images.

## Conflicts of Interest

Kentaro Takezawa is an Editorial Board member of the *International Journal of Urology* and a coauthor of this article. To minimize bias, Kentaro Takezawa was excluded from all editorial decision‐making related to the acceptance of this article for publication.
